# Effect of polymorphisms in porcine guanylate-binding proteins on host resistance to PRRSV infection in experimentally challenged pigs

**DOI:** 10.1186/s13567-020-00745-5

**Published:** 2020-02-19

**Authors:** Amina Khatun, Salik Nazki, Chang-Gi Jeong, Suna Gu, Sameer ul Salam Mattoo, Sim-In Lee, Myun-Sik Yang, Byeonghwi Lim, Kwan-Suk Kim, Bumseok Kim, Kyoung-Tae Lee, Choi-Kyu Park, Sang-Myeong Lee, Won-Il Kim

**Affiliations:** 1 College of Veterinary Medicine, Jeonbuk National University, Iksan, Jeollabuk-do 54596 South Korea; 2 Department of Pathology, Faculty of Animal Science and Veterinary Medicine, Sher-e-Bangla Agricultural University, Dhaka, 1207 Bangladesh; 3 College of Environmental & Biosource Science, Division of Biotechnology, Jeonbuk National University, Iksan, Jeollabuk-do 54596 South Korea; 4 College of Agriculture, Life & Environment Sciences, Department of Animal Science, Chungbuk National University, Cheongju, South Korea; 5 National Institute of Animal Science, Rural Development Administration, Cheonan, South Korea; 6 College of Veterinary Medicine, Kyoungpook National University, Daegu, South Korea

## Abstract

Guanylate-binding proteins (GBP1 and GBP5) are known to be important for host resistance against porcine reproductive and respiratory syndrome virus (PRRSV) infection. In this study, the effects of polymorphisms in GBP1 (GBP1E2 and WUR) and GBP5 on host immune responses against PRRSV were investigated to elucidate the mechanisms governing increased resistance to this disease. Seventy-one pigs [pre-genotyped based on three SNP markers (GBP1E2, WUR, and GBP5)] were assigned to homozygous (*n* = 36) and heterozygous (*n* = 35) groups and challenged with the JA142 PRRSV strain. Another group of nineteen pigs was kept separately as a negative control group. Serum and peripheral blood mononuclear cells (PBMCs) were collected at 0, 3, 7, 14, 21 and 28 days post-challenge (dpc). Viremia and weight gain were measured in all pigs at each time point, and a flow cytometry analysis of PBMCs was performed to evaluate T cell activation. In addition, 15 pigs (5 pigs per homozygous, heterozygous and negative groups) were sacrificed at 3, 14 and 28 dpc, and the local T cell responses were evaluated in the lungs, bronchoalveolar lavage cells (BALc), lymph nodes and tonsils. The heterozygous pigs showed lower viral loads in the serum and lungs and higher weight gains than the homozygous pigs based on the area under the curve calculation. Consistently, compared with the homozygous pigs, the heterozygous pigs exhibited significantly higher levels of IFN-α in the serum, proliferation of various T cells (γδT, Th1, and Th17) in PBMCs and tissues, and cytotoxic T cells in the lungs and BALc. These results indicate that the higher resistance in the pigs heterozygous for the GBP1E2, WUR and GBP5 markers could be mediated by increased antiviral cytokine (IFN-α) production and T cell activation.

## Introduction

Porcine reproductive and respiratory syndrome (PRRS) is the most challenging threat to the swine industry worldwide and is caused by porcine reproductive and respiratory syndrome virus (PRRSV). PRRSV is an enveloped virus with a single-stranded, nonsegmented, positive-sense RNA genome that belongs to the genus *Betaartevirus* of the *Arteriviridae* virus family in *Nidovirales* [1, 2] and has significant impacts on swine production with an annual estimated loss of approximately $664 million in the USA alone [3]. PRRSVs are classified into two genotypes, namely, European (Type 1) and North American (Type 2). No vaccines are currently successful in PRRS control because of the high genetic and antigenic variation among the PRRSV strains [4, 5]. Furthermore, PRRS control via vaccination against highly divergent field strains remains challenging because of the large gaps in our current knowledge regarding PRRSV biology, viral pathogenesis and host immune responses [6, 7]. Therefore, an alternative control measure is essential for PRRS viruses other than vaccination methods. Many previous studies based on genetic tools and methodologies have suggested that genetic improvement in host resistance to PRRSV may provide an alternative opportunity to explore the mechanisms underlying PRRS [8–13]. Thus, insight into the host factors governing PRRS progression and resistance is essential for an understanding and a holistic view of the disease. Many previous studies reported that several genome-wide association studies (GWAS) have identified a quantitative trait locus (QTL) on porcine (*Sus scrofa*) chromosome (SSC) 4 associated with the viral loads and weight gain in pigs infected with PRRSV [14–16]. Among those QTLs, a well-characterized QTL region on SSC4 that is about ½ ~1 Mb in length contains multiple candidate genetic markers, including members of the guanylate-binding protein (GBP) family (GBP1, GBP2, GBP4, GBP5 and GBP6), CCBL2, GTF2B and PKN2, which are associated with pig resistance to PRRSV infection [9, 10, 14, 17]. GBPs are known to have interferon (IFN)-inducible activities and belong to the dynamic superfamily of large GTPases [18, 19]. Similar to interferon (IFN)-inducible GTPase, GBP1 and GBP5 have been previously reported to have substantial antiviral effects against various viruses in humans and mice, including vesicular stomatitis virus (VSV), encephalomyocarditis virus (EMCV), hepatitis C virus (HCV), dengue virus (DENV), human immunodeficiency virus (HIV) and influenza A virus (IAV) [13, 18–25]. Moreover, GBP1 and GBP5 are reportedly critical genes on SSC4 in the pig genome because of their enhanced effects on growth, and these genes play a crucial role in conferring host tolerance and resilience against PRRSV infections [10, 17, 26–29]. GBP1E2 (c.[10A>G; 11A>G]) polymorphisms located in exon-2 cause the replacement of an amino acid (p.Lys4Glu) in the GBP1 gene, which could affect the molecular polarity and influence the protein conformation [13, 26]. The WUR (WUR10000125[A>G]) polymorphism is located immediately upstream of a putative polyadenylation site in the 3′ untranslated region (UTR) of the GBP1 gene and has been demonstrated to be a negative regulator of T cells responses [28]; thus, this polymorphism could be critically involved in the process of protein transcription and translation [14]. In addition, the GBP5 (rs340943904[T>G]) single nucleotide polymorphism (SNP) is a putative causative mutation through alternative splicing that introduces an illegal splice acceptor site in intron-9, which inserts five nucleotides in the GBP5 transcript upstream of exon-10, resulting in a frame shift that could be expected to produce a nonfunctional protein for the GBP5 gene [29]. Previous studies [9, 11, 14, 26, 29] have reported that the SNP tag markers GBP1E2 (c.[10A>G; 11A>G]) and WUR (WUR10000125[A>G]) in GBP1 (hereafter "GBP1E2" and "WUR", respectively) and a SNP (rs340943904[T>G]) in GBP5 (hereafter "GBP5") are important candidate genetic markers for host resistance to PRRS. However, knowledge regarding the roles of these markers in the mechanisms underlying the increased host resistance to PRRSV infection is limited. Therefore, the present study aimed to determine the effects of the GBP1E2 and WUR polymorphisms in GBP1 and GBP5 on the host immune response to PRRSV under experimental conditions to explore the genetic basis of disease resistance and progression in pigs. Accordingly, the effects of polymorphisms in these candidate genes were evaluated in two different ways: (1) effects on pig phenotypes, such as weight gain and viral growth, and (2) effects on host immune responses following PRRSV infection.

## Materials and methods

### Cells and virus

MARC-145 cells, representing an African green monkey kidney cell line known to be highly permissive to PRRSV [30], were used in this study for the viral propagation and assays. The MARC-145 cells were maintained in RPMI growth medium (Gibco^®^ RPMI 1640, Life Technologies, Carlsbad, CA, USA) supplemented with heat-inactivated 10% foetal bovine serum (FBS, Life Technologies), 2 mM l-glutamine, 100× Antibiotic–Antimycotic (Anti-anti, Life Technologies), and a final (1× solution) concentration of 100 IU/mL penicillin, 100 µg/mL streptomycin, and 0.25 µg/mL amphotericin B (Fungizone^®^) at 37 °C in a 5% CO_2_ humidified chamber. JA142, which is a type 2 PRRSV strain, was used in the present study.

### Animal studies

In total, 90 four-week-old pigs (obtained by crossing between Yorkshire female and Landrace male) possessing relatively high genotypic heterogeneity [based on the results of a prescreening for polymorphisms in GBP1 (GBP1E2 and WUR) and GBP5 (GBP5)] were purchased from a PRRSV-negative farm. On arrival, the pigs were randomly housed and divided into two groups of 71 and 19 pigs. After 3 days of acclimatization, all pigs were bled to separate the serum and confirmed to be negative for PRRSV by quantitative real-time reverse transcription PCR (qRT-PCR) (Genetbio, Daejeon, Korea) and enzyme-linked immunosorbent assay (ELISA) (Bionote PRRS Ab ELISA 4.0, Hwasung, Korea). The 71 pigs [pre-genotyped based on three SNP markers (GBP1E2 c10>G, WUR A>G, and GBP5 T>G)] were 100% linkage disequilibrium to one another as follows (Additional file 1) assigned to the homozygous AA/AA/GG animals (*n* = 36) (hereafter “homozygous” or “AA/AA/GG”) and the heterozygous AG/AG/GT animals (n-35) (hereafter “heterozygous” or “AG/AG/GT”). These 71 pigs were challenged with PRRSV (JA142) through the intramuscular (IM) route at a titre of 10^3^ 50% tissue culture infective dose (TCID_50_)/mL (2 mL per pig). The remaining 19 pigs were housed separately and maintained without the virus challenge (hereafter “negative”).

**Figure 1:**
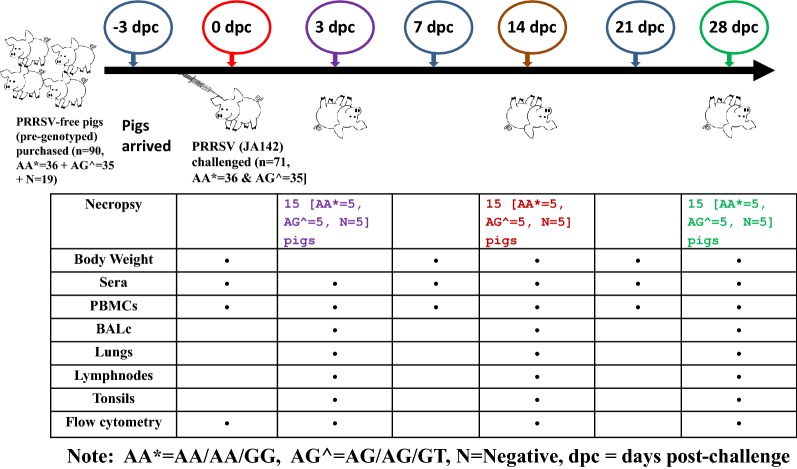
**Design of the animal study.**

The serum was separated at 0 (before challenge), 3, 7, 14, 21 and 28 days post-challenge (dpc) and used to measure the serum viremia, PRRSV-specific antibodies (IgG), virus neutralizing antibodies (VNA), and serum cytokines. Peripheral blood mononuclear cells (PBMCs) were also isolated at 0 (before challenge), 3, 14 and 28 dpc and used for a flow cytometry analysis to evaluate the systemic responses of T cell subsets activated by PRRSV infection. Pigs were also weighed at 0 (before challenge), 7, 14, 21 and 28 dpc until the end of 28 days study period, then average daily weight gain (ADWG) was calculated at every week (i.e. on 7, 14, 21 and 28 dpc). In addition, 15 pigs [5 pigs per group (homozygous, heterozygous and negative) selected randomly] were sacrificed at 3, 14 and 28 dpc. At each necropsy, the bronchoalveolar lavage (BAL), lungs, bronchial lymph nodes and tonsils were collected and used for a flow cytometry analysis to evaluate the local responses of T cell subsets activated by PRRSV infection. All remaining pigs were euthanized for necropsy at 28 dpc, and the pathological evaluation was performed by the same expert pathologist (who was completely blind to the treatment groups) throughout the study period as described previously [31]. Different types of tissues (lungs, bronchial lymph nodes and tonsils) were collected in tubes, snap-frozen using liquid nitrogen and stored immediately at −80 °C until processing. The lung tissues were also collected in 10% neutral-buffered formalin for histopathology. The detailed information related to the animal study is provided in Figure 1. The animal experiment protocol was approved by the Jeonbuk National University Institutional Animal Care and Use Committee (approval number 2016-0043) and performed in accordance with the guidelines and regulations of the committee.

### Virus quantification in the serum

Viral RNA was extracted from the serum samples using a MagMAX™ Viral RNA Isolation Kit (Ambion, Applied Biosystems, Life Technologies, Inc., Carlsbad, CA, USA) according to the manufacturer’s instructions. Then, the viral loads were quantified by qRT-PCR using TaqMan^®^ chemistry based on a previous study [32]. The sequences of the primer and probe were as follows: forward primer: TGTCAGATTCAGGGAGRATAAGTTAC; probe: 6-FAM TGTGGAGTTYAGTYTGCC; and reverse primer: ATCARGCGCACAGTRTGATGC. A one-step qRT-PCR kit (AgPath-ID™ One-Step RT-PCR, Ambion, Applied Biosystems) was used to measure the viral loads in the sera, and a PCR amplification was performed with a 7500 Fast Real-time PCR system (Applied Biosystems, Foster City, CA, USA) according to the manufacturer’s guidelines. The reaction conditions for the one-step qRT-PCR were a total volume of 25 µL, including 5 µL of template RNA, 12.5 µL of 2X RT-PCR buffer, 0.5 µL of each forward and reverse primer (20 pmol, with a final conc. of 0.8 pmol), 0.2 µL of TaqMan^®^ probes (25 pmol, with a final conc. of 1 pmol), 0.5 µL of RNAse inhibitor (40 U/µL; RiboLock™, Thermo Fisher Scientific Inc., Germany), 1 µL of 25X RT-PCR enzyme mix, and 4.8 µL of nuclease-free water. The cycling conditions were as follows: (a) reverse transcription for 10 min at 45 °C; (b) a 10-min activation step at 95 °C; and (c) 40 cycles of 15 s at 95 °C and 45 s at 60 °C. Samples with a threshold cycle (C_t_) of 35 cycles or less were considered positive. A standard curve was generated from known virus titers and used to calculate the amount of PRRSV in each sample by converting the C_t_ value to TCID_50_/mL equivalent values.

### Lung scoring and histopathology

To evaluate the gross and microscopic lung lesions, each lung lobe was scored according to the percentage of lung consolidation and interstitial pneumonia caused by PRRSV infection [31]. The scoring of the microscopic lung lesions was recorded on a scale ranging from 0 to 3 as follows: 0, no lesion; 1, mild interstitial pneumonia; 2, moderate multifocal interstitial pneumonia; and 3, severe interstitial pneumonia.

### Quantification of PRRSV titers in the lungs

The residual virus titres in the lung tissues were measured in MARC-145 cells using a microtitration infectivity assay [33]. Tissue homogenates (10% [weight/volume]) of the lungs were prepared in Dulbecco’s modified Eagle’s medium (DMEM) with antibiotics, vortexed vigorously for 3–5 min, and centrifuged at ~4000 × *g* for 1 h at 4 °C. Then, the supernatant was collected, filtered through a 0.20-µm sterile syringe filter and used as an inoculum to measure the virus titres. The detailed procedure used for the virus titration was based on a previous study [34]. At 5 to 6 days post-inoculation (dpi), the virus titers were measured. The virus titers were calculated based on the cytopathic effect (CPE) and are expressed as TCID_50_/mL [35].

### Detection of anti-PRRSV antibodies

PRRSV-specific antibodies (IgG) were detected in the serum using a commercially available ELISA kit (Bionote PRRS Ab ELISA 4.0, Hwasung, Korea) based on the nucleocapsid protein (NP) according to the manufacturer’s instructions. The S/P ratio (the ratio of the net optical density of the test samples to the net optical density of the positive controls) of the samples was ≥ 0.4, which was considered positive for the PRRSV antibody.

A fluorescent focus neutralization (FFN) assay was performed to detect the virus neutralizing antibody (VNA) titers against JA142 in the serum measured in MARC-145 cells as previously described [36]. The VNA titers are expressed as the reciprocal of the highest dilution in which a 90% or greater reduction in the number of fluorescent focus unit (FFU) was observed compared to the back titers of the respective virus.

### Quantification of cytokines in the serum

The protein levels of the porcine cytokines IFN-α and TNF-α were measured in the pig sera after PRRSV infection by ELISA. The IFN-α protein levels were detected using an in-house ELISA test as previously described [37]. Briefly, 100 μL (1.8 μg/mL) of a mouse anti-pig IFN-α antibody (Clone F17, PBL Assay Science, NJ, USA) were applied as a coating antibody, and a mouse anti-pig IFN-α antibody (Clone K9, PBL Assay Science, NJ, USA) was biotinylated and used as a secondary antibody with recombinant porcine IFN-α (PBL Assay Science, NJ, USA) as a standard. The procedure was carried out using the provided ELISA reagents (eBioscience, CA, USA) following the manufacturer’s instructions. The cytokine TNF-α protein levels were quantified using a commercially available porcine-specific ELISA kit (Porcine TNF-α, DuoSet^®^ ELISA, R&D Systems, MN, USA) according to the manufacturer’s instructions. The results were analysed using SoftMax Pro 5.3 microplate data software (Molecular Devices, CA, USA).

### Isolation of PBMCs, BAL cells (BALc), and mononuclear cells from the lungs, bronchial lymph nodes and tonsils

Blood samples were collected in sodium heparin-containing vacutainers [BD Vacutainer^®^ Sodium Heparin^N^ (NH) 158 USP Units Plus Blood Collection Tubes, BD Biosciences, Franklin Lakes, New Jersey, USA]. Then, the PBMCs were isolated from the blood samples following the density gradient method using Leucosep™ Centrifuge Tubes and Leucoprep™ lymphocyte separation media (Intron Biotechnology, Seongnam-si, Gyeonggi-do, Korea) according to the manufacturer’s instructions. The blood samples were briefly stratified in Leucoprep™ solution at a ratio of 2:1 (blood:Leucoprep) and centrifuged at 1000 × *g* for 10 min. The PBMCs were collected, washed twice and resuspended in fluorescence-activated cell sorting (FACS) buffer [phosphate-buffered saline (PBS) with 3% heat-inactivated foetal bovine (FBS, Gibco, Carlsbad, CA, USA) and 0.02% sodium azide].

The BALc were harvested from the pigs after necropsy based on previous studies [38] with slight modification. Briefly, the pigs were euthanized, and the lungs were aseptically extracted with the trachea and bronchus. After the pathological evaluation, the lungs were lavaged with 50–75 mL of PBS with antibiotics, and the lavaged fluids were collected and centrifuged for 10 min at 1000 × *g*. The resulting supernatants were collected as BAL fluids, while the cell pellets were washed with PBS following treatment with red blood cell (RBC) lysis buffer [eBioscience™ 10× RBC lysis Buffer (Multi-species), Invitrogen by Thermo Fisher Scientific, Life Technologies Corp., Carlsbad, CA, USA]. The washed cell pellet was resuspended in FACS buffer.

Single-cell suspensions were prepared from the lungs, bronchial lymph nodes and tonsils based on previous studies [39], with slight modification. Briefly, the lung and tonsil tissues were collected, washed with sterile PBS, minced, suspended in RPMI medium containing DNase I (25 U/mL) (Sigma-Aldrich, St. Louis, MO, USA) and type II collagenase (2 mg/mL) (Sigma-Aldrich), and incubated for 2 h at 37 °C in a 5% CO_2_ incubator. Then, the digested tissues from the lungs and tonsils were collected, and single-cell suspensions were prepared by grinding with a 40-µm cell strainer (SPL Lifesciences, Pocheon, Gyeonggi-do, South Korea) along with the bronchial lymph nodes; 3 mL of PBS were added, and the samples were mixed by pipetting. Then, the supernatants were collected and centrifuged. The upper supernatant was removed, and the cells pellet was washed with FACS buffer after treatment with RBC lysis buffer. The isolated cell pellets were resuspended in FACS buffer. Finally, the cell numbers and viability of each sample type were evaluated with a Countess™ Automated Cell Counter (Invitrogen, Carlsbad, CA, USA) following the manufacturer’s instructions.

### Flow cytometric analysis

Single-cell suspensions of PBMCs, lungs, BALc, bronchial lymph nodes and tonsils were used for multicolour immunostaining for the flow cytometric analysis. The cells were resuspended in FACS buffer, plated in U-bottom 96-well plates and treated with 2% pig serum (heat inactivated) for 20 min at room temperature (RT) to block the Fc receptors. Then, the cells were stained with appropriate monoclonal antibodies (mAbs) directly conjugated to specific fluorochromes or purified antibodies against pig-specific cell surface markers, followed by other internal staining for FoxP3 and/or the cytokines IL-17 and IFN-γ (Additional file 2). The respective isotype controls were also included in each assay.

Briefly, for the Treg panel (CD4 CD25 FoxP3), two millions cells were first surface stained with PE mouse anti-pig CD4α (Clone 74-12-4; BD Biosciences, Franklin Lakes, New Jersey, USA) and mouse anti-pig CD25 (clone K231.3B2; AbD Serotech, Raleigh, NC, USA) for 30 min on ice in the dark, followed by staining with an allophycocyanin (APC)-conjugated rat anti-mouse IgG1 Ab (Clone RMG1-1; BioLegend, San Diego, CA, USA) as a secondary antibody against CD25. Then, the cells were fixed and permeabilized with FoxP3/Transcription factor staining buffer set (eBioscience) for 30 min on ice and subsequently stained with fluorescein isothiocyanate (FITC) conjugated anti-mouse/rat FoxP3 (Clone FJK-16 s; eBioscience) for 30 min on ice in the dark.

In addition, for the other two panels, i.e., Th1 and Th17 (CD4 IL-17 IFN-γ) and CTL and γδT (CD8 TcR1N4 IFN-γ)], one million cells were treated with 1× cell stimulation cocktail (eBioscience) plus 1x brefeldin A (eBioscience) in RPMI growth medium and incubated for 4–5 h at 37 °C in a 5% CO_2_ humidified chamber. Then, the stimulated cells of the Th1 and Th17 panel were first surface stained with PE mouse anti-pig CD4α (Clone 74-12-4; BD Biosciences) for 30 min on ice in the dark. Then, the cells were fixed and permeabilized with Intracellular Fixation and Permeabilization buffer set (eBioscience) for 30 min on ice, followed by staining with anti-human IL-17A APC (clone eBio64DEC17; eBioscience) and PerCP-Cy™ 5.5 mouse anti-pig IFN-γ (Clone P2G10; BD Biosciences) for 30 min on ice in the dark. Similarly, the stimulated cells of the CTLs and γδT panel were also first surface stained with FITC mouse anti-pig CD8α (Clone 76-2-11; BD Biosciences) and mouse anti-pig TcR1N4 [anti-swine TCR1 δ chain specific (Clone PGBL22A, Kingfisher Biotech. Inc., MN, USA)] for 30 min on ice in the dark, followed by staining with an APC-conjugated rat anti-mouse IgG1 Ab (Clone RMG1-1; BioLegend) as a secondary antibody against TcR1N4. Subsequently, the cells were fixed and permeabilized with intracellular fixation and permeabilization buffer set (eBioscience) for 30 min on ice, followed by staining with PerCP-Cy™ 5.5 mouse anti-pig IFN-γ (BD Biosciences).

Finally, the stained cells in all panels were resuspended in 100 μL of cold FACS buffer, and flow cytometry was performed using an Accuri C6 flow cytometer (BD Accuri™ C6 Plus, BD Biosciences). The phenotype data for 100 000 events were collected, followed by a gating strategy based on forward scatter (FSC) and side scatter (SSC), and the data were analysed using BD Accuri™ C6 Plus software version 1.0.23.1 (BD Biosciences) based on the marker expression on the cell surface in the appropriate gate as previously described [40].

### Data analysis

The association between the effects of the polymorphisms in the candidate genes and the immune phenotypes was tested using all data generated from the PRRSV challenged pigs (*n* = 71). The correlation (Spearman’s) analysis between two parameters was performed by a linear regression. Since the data did not display Gaussian distribution based on Shapiro-Wilks Normality test, a nonparametric *t*-test (Mann–Whitney *U* test) was used to compare the significant differences within specific two genotypes/or groups employed for each parameter, such as the viral loads in the serum and lung tissues, average daily weight gain (ADWG), responses of antibodies and cytokine protein levels and phenotypes of activated T cell subsets. The differences were considered statistically significant at *p* < 0.05. GraphPad Prism 5.0.2 (GraphPad Software, Inc., CA, USA) was used to generate the graphs, and the statistical analysis was performed using SPSS Advanced Statistics 17.0 software (SPSS, Inc., Chicago, USA).

## Results

### Associations between the average daily weight gain (ADWG) and viremia

**Figure 2:**
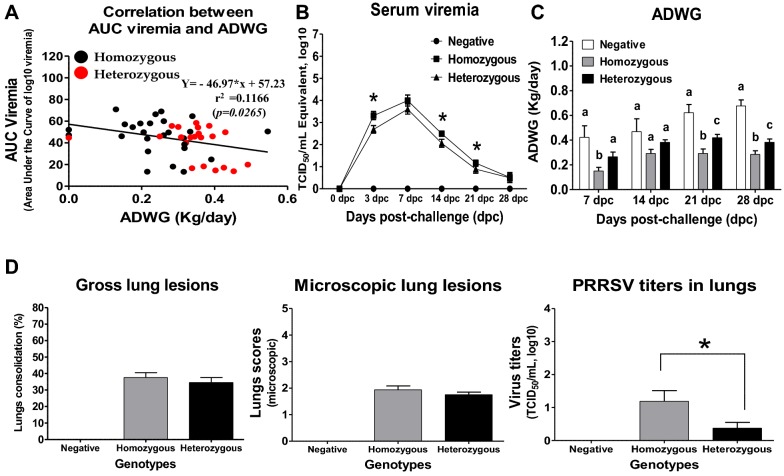
**Correlation between AUC (area under the curve) viremia and ADWG, AUC viremia and PRRSV viral loads in the serum and lungs, and ADWG. A** The correlation (Spearman’s, r) between AUC viremia (0–21 dpc) and ADWG (until 28 dpc) was tested in the pigs after infection with JA142. **B** The viral loads in the serum samples collected from all pigs were quantified (using qRT-PCR) at 0 (before challenge), 3, 7, 14, 21 and 28 dpc. The viral loads are expressed as TCID_50_ equivalent/mL (log_10_). **C** ADWG in all pigs was calculated at every week up to 28 dpc and expressed as kg/day. **D** The lungs pathology (gross and microscopic lesions), and residual virus titers in the lung tissues collected from pigs at 28 dpc were measured using a microtitration infectivity assay performed in MARC-145 cells and presented as TCID_50_/mL (log_10_). The error bars represent the standard error of the mean (SEM). Asterisks indicate significant differences in each parameter among the genotypes (*p < 0.05). The bars showing different letters represent the values that differ significantly from each other (*p* < 0.05).

The impact of PRRSV was investigated in pigs following infection by measuring the average daily weight gain (ADWG) and AUC (area under the curve) viremia until 21 dpc (Figure 2). The pigs in the negative group exhibited 0.464 ± 0.239 (mean ± SD) kg/day of ADWG and remained negative for PRRSV until the end of the experiment. However, the pigs challenged with JA142 exhibited 0.286 ± 0.119 kg/day of ADWG and average viremia with virus titers of 10^2.287^ ± 10^0.996^ TCID_50_ (equivalent)/mL, while viremia was sharply elevated to the peak virus titers at 7 dpc and gradually declined up to 28 dpc. As expected, the viremia following PRRSV infection exerted significant negative effects on the pigs’ growth rate and was negatively correlated with the ADWG (r = −0.3236, *p* = 0.0265) (Figure 2A).

### Associations between the polymorphisms in the candidate genes and viral loads in the sera

The polymorphisms in the candidate genes were significantly related to serum viremia in the pigs following the PRRSV (JA142) infection. The heterozygous pigs exhibited significantly lower levels of viremia than the homozygous pigs throughout the study period (Figure 2B). These heterozygous pigs had average viremia with virus titers of 10^2.14^ ± 10^0.57^ TCID_50_ (equivalent)/mL, which was significantly lower (*p* < 0.0003) than the titers of 10^2.86^ ± 10^0.72^ TCID_50_ (equivalent)/mL observed in the homozygous pigs (Figure 2B).

### Associations between genotypes and growth traits

The effects of the polymorphisms in the candidate genes on the pigs’ weight gain (WG) were investigated at every week up to 28 dpc following experimental infection with PRRSV. As summarized in Figure 2C, the results showed that the polymorphisms in the candidate genes were highly associated with the ADWG measured in the pigs after the JA142 infection. The heterozygous pigs exhibited an ADWG of 0.34 ± 0.10 kg/day, which was significantly higher (*p* < 0.0001) than the 0.23 ± 0.11 kg/day ADWG observed in the homozygous pigs.

### Lung pathology and residual PRRSV titers

The gross and microscopic lungs scores were recorded according to a previous study [31]. No significant differences were observed in the lung scores (gross and microscopic) between the heterozygous and homozygous pigs (Figure 2D).

The residual viral loads in the lung tissues were measured in MARC-145 cells. The results showed that the heterozygous pigs had average virus titers of 10^0.37^ ± 10^0.86^ TCID_50_/mL, which were significantly lower (*p* < 0.05) than the titers of 10^1.19^ ± 10^1.60^ TCID_50_/mL observed in the homozygous pigs as measured in the lung tissues at 28 dpc (Figure 2D).

### Associations between the genotypes and the levels of antibody responses following PRRSV infection

**Figure 3:**
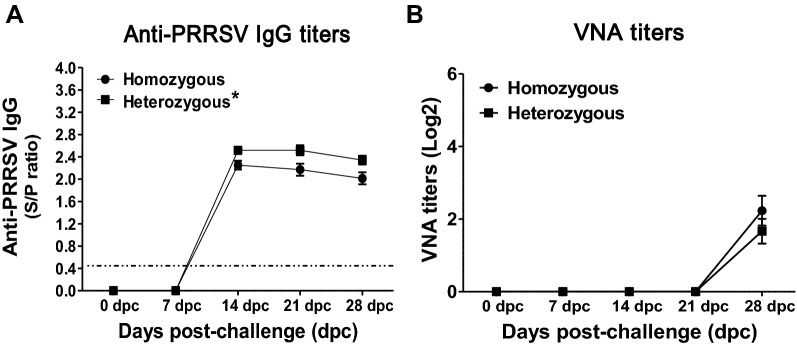
**Quantification of anti-PRRSV antibodies (IgG) and VNA titers measured in PRRSV-infected pigs. A** Anti-PRRSV antibody (IgG) response was measured in serum samples collected weekly until 28 dpc using a nucleocapsid (N) protein-based ELISA kit based on the manufacturer’s instructions, and the result is expressed as the S/P ratio. An S/P ratio ≥ 0.4 is considered positive for PRRSV-specific antibodies (IgG). **B** The levels of the VNA titers were measured using a fluorescent focus neutralization (FFN) assay. The error bars represent the SEM. Asterisks indicate significant differences in the level of anti-PRRSV IgG among the genotypes (*p < 0.05).

The association between the genotypes and the level of induced antibodies (IgG) was evaluated in pigs following PRRSV infection by ELISA based on the nucleocapsid protein (NP). All infected pigs of both genotypes (heterozygous and homozygous) became seropositive at 14 dpc, which was maintained up to 28 dpc, and the pigs in the negative group were seronegative throughout the study period. However, the heterozygous pigs exhibited a higher S/P ratio than the homozygous pigs (Figure 3A).

The virus neutralizing antibody (VNA) titers were also measured in the pigs’ serum against JA142 following infection. The VNA responses were delayed and usually detected at a later stage of infection with low titers ≥ 2 (log2) at 28 dpc. However, no significant difference was observed in the levels of induced VNA titers between the heterozygous and homozygous pigs (Figure 3B).

### Associations between the genotypes and cytokine protein levels in the pigs’ serum following PRRSV infection

**Figure 4:**
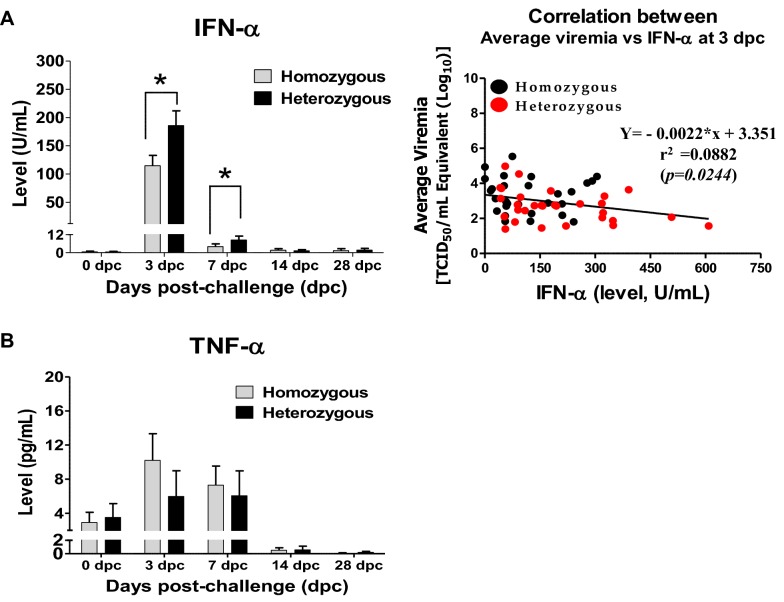
**Quantification of the cytokine protein levels in serum collected from PRRSV-infected pigs.** Serum samples collected at 0, 3, 7, 14, and 28 dpc were analysed to detect porcine cytokines. **A** IFN-α by ELISA and a correlation with average viremia at 3 dpc. **B** TNF-α by ELISA. The error bars represent the SEM. Asterisks indicate significant differences in the levels of cytokines induced by PRRSV among the genotypes (*p < 0.05).

The associations between the polymorphisms in the candidate genes and the cytokine protein levels were quantified in the serum using ELISA. The heterozygous pigs exhibited significantly higher levels of IFN-α in the serum than the homozygous pigs between 3 and 7 dpc with a maximum mean value of 185.8 U/mL as measured at 3 dpc compared with the mean value of 114.9 U/mL observed in the homozygous pigs, which was dramatically reduced from 7 dpc in both genotypes (Figure 4A). Furthermore, a correlation analysis was performed to see the association between the innate immune response and viral growth following PRRSV infection in pigs at 3 dpc between the level of induced IFN-α in serum and average viremia, respectively. As expected, PRRS virus replication had a significantly negative correlation with the level of induced IFN-α response in pigs (r = −0.2954, *p* = 0.0244) (Figure 4A). In contrast, the response of the induced TNF-α levels following infection was very low, and no significant difference was observed between the genotypes (Figure 4B).

### Associations between the genotypes and T cell responses in PBMCs following PRRSV infection

**Figure 5:**
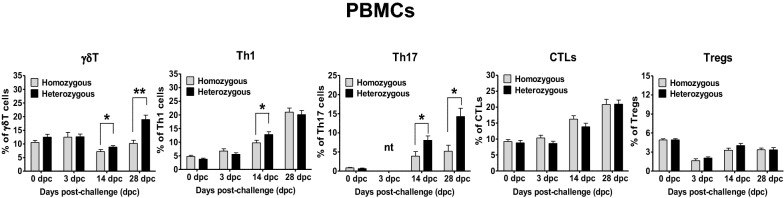
**T cell responses in PBMCs isolated from pigs after PRRSV infection.** PBMCs were separated at the indicated days, immunostained with the appropriate antibodies, and analysed by flow cytometry to detect gamma-delta (γδ) T cells (γδT), type 1 helper T cells (Th1), T helper 17 cells (Th17), cytotoxic T lymphocytes (CTLs) and regulatory T cells (Tregs). The error bars represent the SEM. Asterisks indicate significant differences in T cell proliferation among the genotypes (*p < 0.05; **p < 0.001). *nt: not tested because of sample shortage.

The associations between the polymorphisms in the candidate genes and the T cell responses were evaluated in PBMCs (Figure 5). Compared with the homozygous pigs, the heterozygous pigs exhibited significantly higher numbers of *γδ*T (CD8^+^ TcR1N4^+^) cells at 14 and 28 dpc, Th1 (CD4^+^IFN-γ^+^) cells at 14 dpc, and Th17 (CD4^+^IL-17^+^) cells at 14 and 28 dpc. However, no significant differences in the numbers of activated CTLs (CD8^+^IFN-γ^+^) and/or Tregs (CD4^+^CD25^+^FoxP3^+^) were observed in the pigs between the genotypes.

### Associations between the genotypes and T cell responses in various tissues after PRRSV infection

**Figure 6:**
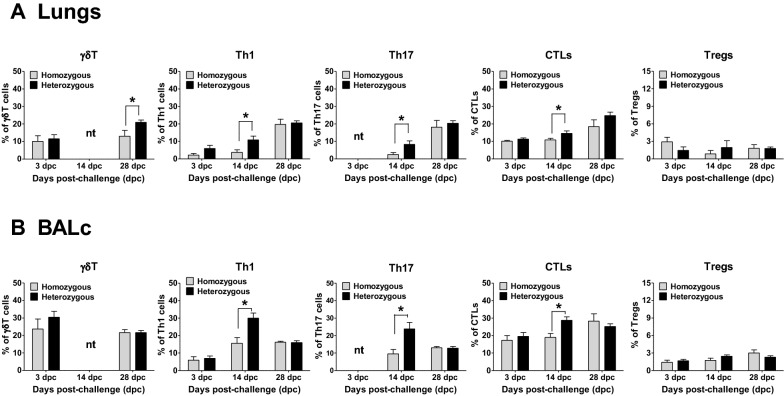
**T cell responses in lungs and BALc collected from pigs after PRRSV infection.** Mononuclear cells isolated from the lungs (**A**) and BALc (**B**) were immunostained with the appropriate antibodies on the indicated days and then analysed by flow cytometry to detect gamma-delta (γδ) T cells (γδT), type 1 helper T cells (Th1), T helper 17 cells (Th17), cytotoxic T lymphocytes (CTLs) and regulatory T cells (Tregs). The error bars represent the SEM. Asterisks indicate significant differences in T cell proliferation among the genotypes (*p < 0.05). *nt: not tested because of sample shortage.

**Figure 7:**
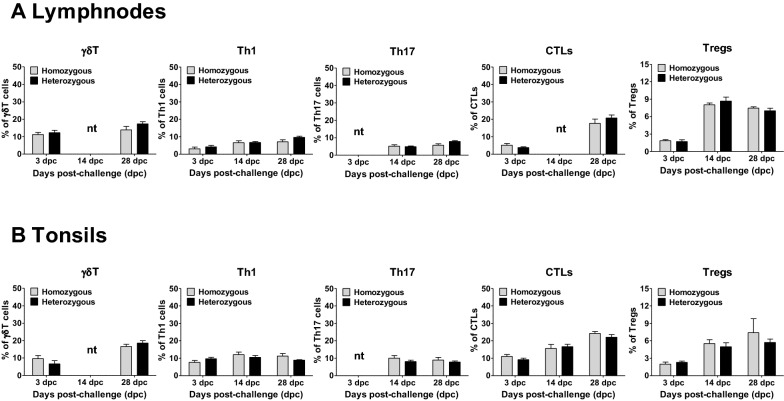
**T cell responses in lymph nodes and tonsils collected from pigs after PRRSV infection.** Mononuclear cells isolated from the lymph nodes (**A**) and tonsils (**B**) were immunostained with the appropriate antibodies on the indicated days and analysed by flow cytometry to detect gamma-delta (γδ) T cells (γδT), type 1 helper T cells (Th1), T helper 17 cells (Th17), cytotoxic T lymphocytes (CTLs) and regulatory T cells (Tregs). The error bars represent the SEM. *nt: not tested because of sample shortage.

The associations between the polymorphisms in the candidate genes and the T cells responses were further analysed at the sites of PRRSV infection in the lungs, BALc, and other lymphoid organs, such as the lymph nodes and tonsils. As summarized in Figure 6, the heterozygous pigs exhibited significantly higher numbers of *γδ*T cells in the lungs at 28 dpc (Figure 6A). Similarly, these heterozygous pigs exhibited higher numbers of Th1 cells, Th17 cells and CTLs in both the lungs and BALc than the homozygous pigs at 14 dpc (Figures 6A, B), although no significant difference was observed in the numbers of Tregs between the genotypes (Figure 6). In contrast, no significant differences were observed in the numbers of T cell subsets in the lymph nodes and tonsils between the heterozygous and homozygous pigs (Figure 7).

## Discussion

**Figure 8:**
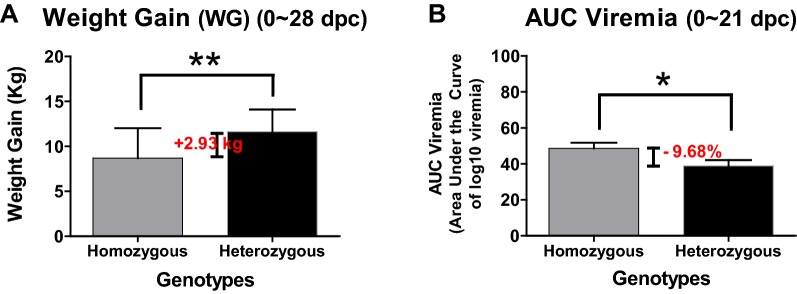
**Measurement of weight gain and AUC (area under the curve) viremia in pigs following PRRSV infection. A** Weight gain (WG) was calculated between 0 to 28 dpc and expressed in Kg. **B** AUC (area under the curve) viremia (of log10 transformed viremia) was measured between 0 to 21 dpc.

Although GBP1 and GBP5 have been demonstrated to be highly associated with increased host resistance against infection with different PRRSV strains [9, 11, 14, 16, 29], the mechanisms of the increased host resistance to PRRS have not been characterized to date. Hence, the present study evaluated the effects of the GBP1E2, WUR and GBP5 polymorphisms on host immune responses against PRRSV infection to explore the possible mechanisms underlying the increased resistance to the disease. Based on the results presented in this study, it was concluded that polymorphisms in the candidate genes (GBP1E2, WUR and GBP5) were significantly associated with host resistance to PRRS after experimental infection with the JA142 PRRSV strain. Boodicker et al. [11] reported that resilient (heterozygous) pigs had a significant reduction (5%) in viremia levels [area under the curve (AUC) up to 21 dpc] and gained 2 kg more weight at 42 dpc as compared to susceptible (homozygous) pigs. Similarly, in the current study, heterozygous pigs had a significant reduction (9.68%) in AUC viremia levels and gained 2.93 kg more weight at 28 dpc as compared to susceptible (homozygous) pigs (Figure 8). Compared to the homozygous pigs, the heterozygous pigs exhibited reduced viral loads in the serum and lungs after the challenge with PRRSV (Figures 2B, D). These results are also consistent with the results reported in previous studies in which pigs heterozygous for the GBP1E2 [26] and WUR [10, 14, 41, 42] genotypes of GBP1 showed significantly lower viral loads following PRRSV infection and/or vaccination and even during coinfection with PRRSV and PCV2b. As previously reported [29, 43], a causal mutation in the GBP5 gene was predicted to produce a nonfunctional protein for GBP5 in homozygous pigs. The functional impacts of this truncated GBP5 protein on host responses to PRRSV infection must be explored [10]. In addition, Schroyen et al. [43] reported that homozygous GBP5 (GG) encodes the truncated version of GBP5, which is unable to bind phosphoinositide 3 kinase (PI3K) and, thus, favours PRRSV entry and replication compared to the heterozygous genotype as demonstrated in previous animal experiments [10, 29, 43]. The PI3K-Akt pathway is critically involved in the virus entry of many viruses [44–46], including PRRSV [47, 48]. Moreover, GBP5 reportedly impairs the infectivity of HIV-1 and IAV [10, 23, 24]. Consistently, compared to the homozygous pigs, the pigs heterozygous for the GBP1E2, WUR and GBP5 SNP sequences exhibited significantly increased ADWG after infection with PRRSV (Figure 2C), which is also supported by previous studies investigating GBP1E2 [26] and WUR [14, 41, 42], although no significant effect was found when the pigs were co-infected with PCV2b [41].

PRRSV is a poor inducer of the innate antiviral cytokines IFN-α and TNF-α, and their levels remain low as demonstrated in the infected pigs, which eventually causes a weak and delayed induction of adaptive immune responses, such as neutralizing antibody and T cell responses [7, 49–52]. In this study, compared to the homozygous pigs, the heterozygous pigs exhibited significantly higher levels of IFN-α in the serum between 3 and 7 dpc, followed by a rapid decline. This result may suggest that the pigs with the heterozygous genotype could potentially have enhanced IFN-α production following PRRSV infection (Figure 4A), which may also be consistently related with the reduced viral loads (Figure 4A) and increased ADWG observed in the heterozygous pigs compared with those observed in the homozygous pigs as described above (Figure 2C). However, no significant difference in the levels of TNF-α was observed between the heterozygous and homozygous pigs (Figure 4B).

T cell-mediated immune responses play a crucial role in enhancing the protective immunity against PRRSV infection [53–55]. In this study, the effect of the polymorphisms in the candidate genes on T cells responses was evaluated following PRRSV infection. GBP1 is reportedly a known regulator of T cell activation [56]. Notably, we found that the heterozygous pigs had significantly higher numbers of T cell subsets (γδT, Th1, and Th17 cells) in the PBMCs, lungs and BALc than the homozygous pigs (Figures 5 and 6). However, these heterozygous pigs also had significantly higher numbers of CTLs in the lungs and BALc but not in the PBMCs compared to the homozygous pigs (Figures 5 and 6), which may suggest that CTLs response in the lungs rather than in PBMCs could be more important for PRRSV clearance in heterozygous pigs. These results are supported by the previous studies reported that CTLs or T cells mediated immune responses are critical for viral clearance against PRRSV and other viral infections as well [57–59]. In fact, the viral loads in the lungs of the heterozygous pigs were also significantly lower than those in the lungs from the homozygous pigs, although no significant differences were observed in the gross and microscopic lung lesions between the genotypes (Figure 2D).

PRRSV infection in pigs is characterized by the early induction of non-neutralizing antibodies, followed by the delayed induction of neutralizing antibodies (NAbs) [4, 60]. However, the role of non-neutralizing antibodies in PRRSV infection remains unknown [49, 61], although these antibodies reportedly confer some clinical protection in other viral infections [62–64]. In the current study, all infected pigs exhibited non-neutralizing antibodies (IgG) based on the PRRSV-nucleocapsid protein at 14 dpc, which was detected until 28 dpc, while the heterozygous pigs showed a higher S/P ratio than the homozygous pigs (Figure 3A). Neutralizing antibodies (NAbs) play a critical role in the immunological control of a wide variety of viral infections [65]. However, the importance of NAbs in PRRSV infections is not completely understood [49, 61] as they usually appear after 3–4 weeks of infection, and due to their low titers, they are ineffective in the clearance of the virus [4, 61, 66, 67]. Consistently, we found that the virus NAbs (VNA) response was delayed, and low titers ≥ 2 (log2) were detected at 28 dpc when viremia was almost resolved (Figure 2B), which can also be supported by previous studies reporting that VNA do not play a protective role in PRRSV infection [4, 49, 61, 66, 67].

In conclusion, GBP1E2, WUR and GBP5, which are the most important genetic markers located on swine chromosome 4 (SSC4), were significantly associated with host resistance to PRRS, and the possible mechanisms of their increased resistance to PRRSV infection could be mediated by the enhanced induction of antiviral cytokines (IFN-α) and the increased T cell mediated immune response in pigs.

## Supplementary information

**Additional file 1. Pigs with SNP genotypes for GBP1E2 and WUR in the GBP1 and GBP5 genes used in the present study.**

**Additional file 2. Information regarding the antibodies used for the FACS staining in the present study.**

## Data Availability

All data generated or analysed during the study are included in this published article. The datasets used and/or analysed during the present research project are available from the corresponding author upon reasonable request.

## Abbreviations

GBP: guanylate-binding proteins
PRRSV: porcine reproductive and respiratory syndrome virus
PRRS: porcine reproductive and respiratory syndrome
SNP: single nucleotide polymorphism
PBMCs: peripheral blood mononuclear cells
IFN-α: interferon-alpha
MLV: modified-live virus
GWAS: genome-wide association studies
QTL: quantitative trait loci
SSCs: porcine (*Sus scrofa*) chromosomes
VSV: vesicular stomatitis virus
EMCV: encephalomyocarditis virus
HCV: hepatitis C virus
DENV: dengue virus
HIV: human immunodeficiency virus
IAV: influenza A virus
qRT-PCR: quantitative real-time reverse transcription PCR
ELISA: enzyme-linked immunosorbent assay
VNA: virus neutralizing antibodies
BAL: bronchoalveolar lavage
DMEM: Dulbecco’s modified Eagle’s medium
CPE: cytopathic effect
FFN: fluorescent focus neutralization
FFU: fluorescent focus unit
TNF-α: tumor necrosis factor-alpha
BALc: bronchoalveolar lavage cells
FACS: fluorescence-activated cell sorting
mAbs: monoclonal antibodies
IL-17: interleukin-17
FoxP3: forkhead box P3
APC: allophycocyanin
IFN-γ: interferon-gamma
ADWG: average daily weight gain
dpc: days post-challenge
FITC: fluorescein isothiocyanate
RBC: red blood cell
TCID: tissue culture infective dose
PBS: phosphate-buffered saline
γδT: gamma delta T cell
Th1: type 1 T helper cell
Th17: T-helper cell 17
CTLs: cytotoxic T lymphocytes
Treg: regulatory T cell
