# Supplementary material for: Effect of polymorphisms in porcine guanylate-binding proteins on host resistance to PRRSV infection in experimentally challenged pigs
Source: Vet Res. 2020 Feb 19;51:14. doi: 10.1186/s13567-020-00745-5 (PMC7031929; doi:10.1186/s13567-020-00745-5)
Supplement: Supplementary file 1 — Additional file 1. Pigs with SNP genotypes for GBP1E2 and WUR in the GBP1 and GBP5 genes used in the present study. [file 13567_2020_745_MOESM1_ESM.docx]

**Additional file 1: Pigs with SNP genotypes for GBP1E2 and WUR in GBP1 and GBP5 genes used in the present study**

| Pig # | SNP Genotypes | | | Pig # | SNP Genotypes | | | Pig # | SNP Genotypes | | |
| --- | --- | --- | --- | --- | --- | --- | --- | --- | --- | --- | --- |
|  | **GBP1E2** | **WUR** | **GBP5** |  | **GBP1E2** | **WUR** | **GBP5** |  | **GBP1E2** | **WUR** | **GBP5** |
| 1 | AA | **AA** | **GG** | **37** | AG | **AG** | **GT** | **73** | AG | **AG** | **GT** |
| 2 | AA | **AA** | **GG** | **38** | AG | **AG** | **GT** | **74** | AG | **AG** | **GT** |
| 3 | AA | **AA** | **GG** | **39** | AG | **AG** | **GT** | **75** | AG | **AG** | **GT** |
| 4 | AA | **AA** | **GG** | **40** | AG | **AG** | **GT** | **76** | AG | **AG** | **GT** |
| 5 | AA | **AA** | **GG** | **41** | AG | **AG** | **GT** | **77** | AG | **AG** | **GT** |
| 6 | AA | **AA** | **GG** | **42** | AG | **AG** | **GT** | **78** | AG | **AG** | **GT** |
| 7 | AA | **AA** | **GG** | **43** | AG | **AG** | **GT** | **79** | AG | **AG** | **GT** |
| 8 | AA | **AA** | **GG** | **44** | AG | **AG** | **GT** | **80** | AG | **AG** | **GT** |
| 9 | AA | **AA** | **GG** | **45** | AG | **AG** | **GT** | **81** | AG | **AG** | **GT** |
| 10 | AA | **AA** | **GG** | **46** | AG | **AG** | **GT** | **82** | AG | **AG** | **GT** |
| 11 | AA | **AA** | **GG** | **47** | AG | **AG** | **GT** | **83** | AG | **AG** | **GT** |
| 12 | AA | **AA** | **GG** | **48** | AG | **AG** | **GT** | **84** | AG | **AG** | **GT** |
| 13 | AA | **AA** | **GG** | **49** | AG | **AG** | **GT** | **85** | AG | **AG** | **GT** |
| 14 | AA | **AA** | **GG** | **50** | AG | **AG** | **GT** | **86** | AG | **AG** | **GT** |
| 15 | AA | **AA** | **GG** | **51** | AG | **AG** | **GT** | **87** | AG | **AG** | **GT** |
| 16 | AA | **AA** | **GG** | **52** | AG | **AG** | **GT** | **88** | AG | **AG** | **GT** |
| 17 | AA | **AA** | **GG** | **53** | AG | **AG** | **GT** | **89** | AG | **AG** | **GT** |
| 18 | AA | **AA** | **GG** | **54** | AG | **AG** | **GT** | **90** | AG | **AG** | **GT** |
| 19 | AA | **AA** | **GG** | **55** | AG | **AG** | **GT** |  |  |  |  |
| 20 | AA | **AA** | **GG** | **56** | AG | **AG** | **GT** |  |  |  |  |
| 21 | AA | **AA** | **GG** | **57** | AG | **AG** | **GT** |  |  |  |  |
| 22 | AA | **AA** | **GG** | **58** | AG | **AG** | **GT** |  |  |  |  |
| 23 | AA | **AA** | **GG** | **59** | AG | **AG** | **GT** |  |  |  |  |
| 24 | AA | **AA** | **GG** | **60** | AG | **AG** | **GT** |  |  |  |  |
| 25 | AA | **AA** | **GG** | **61** | AG | **AG** | **GT** |  |  |  |  |
| 26 | AA | **AA** | **GG** | **62** | AG | **AG** | **GT** |  |  |  |  |
| 27 | AA | **AA** | **GG** | **63** | AG | **AG** | **GT** |  |  |  |  |
| 28 | AA | **AA** | **GG** | **64** | AG | **AG** | **GT** |  |  |  |  |
| 29 | AA | **AA** | **GG** | **65** | AG | **AG** | **GT** |  |  |  |  |
| 30 | AA | **AA** | **GG** | **66** | AG | **AG** | **GT** |  |  |  |  |
| 31 | AA | **AA** | **GG** | **67** | AG | **AG** | **GT** |  |  |  |  |
| 32 | AA | **AA** | **GG** | **68** | AG | **AG** | **GT** |  |  |  |  |
| 33 | AA | **AA** | **GG** | **69** | AG | **AG** | **GT** |  |  |  |  |
| 34 | AA | **AA** | **GG** | **70** | AG | **AG** | **GT** |  |  |  |  |
| 35 | AA | **AA** | **GG** | **71** | AG | **AG** | **GT** |  |  |  |  |
| 36 | AA | **AA** | **GG** | **72** | AA | **AA** | **GG** |  |  |  |  |

**Note**: Pigs # **1~36** = **Homozygous** group, #**37~71** = **Heterozygous** group, and #**72~90** = **Negative** group
