# Supplementary material for: Effect of polymorphisms in porcine guanylate-binding proteins on host resistance to PRRSV infection in experimentally challenged pigs
Source: Vet Res. 2020 Feb 19;51:14. doi: 10.1186/s13567-020-00745-5 (PMC7031929; doi:10.1186/s13567-020-00745-5)
Supplement: Supplementary file 2 — Additional file 2. Information regarding the antibodies used for the FACS staining in the present study. [file 13567_2020_745_MOESM2_ESM.docx]

**Additional file 2: Antibodies used for FACS staining in each panel.**

| **FACS**  **Staining Panels** | **Antigen** | **Clone** | **Isotype** | **Fluorochrome** | **Labeling strategy** | **Source of**  **primary Antibodies (Abs)** |
| --- | --- | --- | --- | --- | --- | --- |
| CD4 CD25 FoxP3 | CD4α | 74-12-4 | IgG2b | PE | directly conjugated | BD Biosciences |
|  | CD25 | K231.3B2 | IgG1 | APC | secondary antibody**^a^** | AbD Serotech |
|  | FoxP3 | FJK-16 s | IgG2a | FITC | directly conjugated | eBioscience |
| CD4 IFN-γ IL-17 | CD4α | 74-12-4 | IgG2b | PE | directly conjugated | BD Biosciences |
|  | IFN- γ | P2G10 | IgG1 | PercpCy5.5 | directly conjugated | BD Biosciences |
|  | IL-17A | eBio64DEC17 | IgG1 | APC | directly conjugated | eBioscience |
| CD8 TcR1N4 IFN-γ | CD8α | 76-2-11 | IgG2a | FITC | directly conjugated | BD Biosciences |
|  | TcR1N4 | PGBL22A | IgG1 | APC | secondary antibody**^a^** | Kingfisher Biotech. Inc. |
|  | IFN- γ | P2G10 | IgG1 | PercpCy5.5 | directly conjugated | BD Biosciences |

**^a^** Rat anti-mouse IgG1-APC, Clone RMG1-1, Biolegend.
